# Dietary Supplement Enriched in Antioxidants and Omega-3 Protects from Progressive Light-Induced Retinal Degeneration

**DOI:** 10.1371/journal.pone.0128395

**Published:** 2015-06-04

**Authors:** Khaoula Ramchani-Ben Othman, Christine Cercy, Mohamed Amri, Michel Doly, Isabelle Ranchon-Cole

**Affiliations:** 1 Université Auvergne, UFR Pharmacie, Laboratoire de Biophysique Neurosensorielle, Inserm UMR 1107, Clermont-Ferrand, France; 2 Department of Biological Sciences, Tunis El Manar University, Laboratory of Functional Neurophysiology and Pathology, UR/11ES09, El Manar 1, Tunis, Tunisia; Univeristy of Miami, UNITED STATES

## Abstract

In the present study, we have evaluated one of the dietary supplements enriched with antioxidants and fish oil used in clinical care for patient with age-related macular degeneration. Rats were orally fed by a gastric canula daily with 0.2 ml of water or dietary supplement until they were sacrificed. After one week of treatment, animals were either sacrificed for lipid analysis in plasma and retina, or used for evaluation of rod-response recovery by electroretinography (ERG) followed by their sacrifice to measure rhodopsin content, or used for progressive light-induced retinal degeneration (PLIRD). For PLIRD, animals were transferred to bright cyclic light for one week. Retinal damage was quantified by ERG, histology and detection of apoptotic nuclei. Animals kept in dim-cyclic-light were processed in parallel. PLIRD induced a thinning of the outer nuclear layer and a reduction of the b-wave amplitude of the ERG in the water group. Retinal structure and function were preserved in supplemented animals. Supplement induced a significant increase in omega-3 fatty acids in plasma by 168% for eicosapentaenoic acid (EPA), 142% for docosapentaenoic acid (DPA) and 19% for docosahexaenoic acid (DHA) and a decrease in the omega-6 fatty acids, DPA by 28%. In the retina, supplement induced significant reduction of linolenic acid by 67% and an increase in EPA and DPA by 80% and 72%, respectively, associated with significant decrease in omega-6 DPA by 42%. Supplement did not affect rhodopsin content or rod-response recovery. The present data indicate that supplement rapidly modified the fatty acid content and induced an accumulation of EPA in the retina without affecting rhodopsin content or recovery. In addition, it protected the retina from oxidative stress induced by light. Therefore, this supplement might be beneficial to slow down progression of certain retinal degeneration.

## Introduction

In patient with early age-related macular degeneration (AMD), supplementation with vitamins and trace elements has become standard in clinical care. This is based to a large degree on the results of the Age-Related Eye Disease Study 1 (AREDS 1), which proved that a food supplement containing vitamin C, vitamin E, β-carotene, and zinc reduces the risk of developing late-stage AMD in high-risk patients by approximately 25% over a period of more than 6 years [1]. However, since the results of AREDS 1 were published, a number of concerns regarding the included components and their dosing have been raised [2–4]. As such, the AREDS 2 was launched, and investigated as part of the primary randomization whether the addition of either lutein/zeaxanthin or omega-3 free fatty acids or a combination of lutein/zeaxanthin and omega-3 free fatty acids exerts an additional effect to the AREDS 1 formulation [5].

Given the high prevalence of AMD in the elderly and the enormous socioeconomic burden of the disease, dietary supplement for ocular purpose have exploded on the market containing the ingredients of the AREDS 1 and AREDS 2 formulations in modified dosing, but also including ginkgo biloba, resveratrol, flavonoids, taurine, aronia extract, or alipoic acid based on their antioxidative properties. In addition, unlike new drugs, dietary supplements are not reviewed and approved by FDA (Food and drug agency, USA) or EMA (European Medicine Agency, Europe) based on their safety and effectiveness. The Food Supplements Directive (FSD) Directive 2002/46/EC, has only established a list of allowable vitamins and minerals, and sets labeling requirements. The Directive calls for the establishment of harmonized minimum and maximum dosage amounts however this has yet to be done. Moreover, substances other than vitamins and minerals are not covered by the directive. Therefore, dietary supplements do not need individual marketing authorization based on evaluation by experts of a record submitted by the industrial who wants to market them. However, mechanism by which complex formulation protects the retina has not been investigated in-vivo.

In this context, the aim of the present study was to test one of these dietary supplements in an in-vivo model of retinal degeneration. In this model, retinal degeneration is caused by progressive light-induced damage (phototoxicity). First, this model in animals has been extensively used to evaluate neuroprotective effects of molecules in retinal degenerations such as AMD because it presents similar mechanism to most of human retinal degeneration such as apoptosis, oxidative stress and inflammation [6,7]. Second, increasing evidence suggest that light-injury to the retina accelerates certain retinal degeneration [8–10] and phototoxicity becomes a serious consideration in the presence of retinal disease [11–14].

In the present study, we have first evaluated the protective effect of the dietary supplement against light-induced retinal degeneration. Second, in order to better understand how it protects the retina, we have investigated the effect of daily supplementation on fatty acid composition of plasma and retina, on rhodopsin content and recovery after bleaching.

## Materials and Methods

### Ethics Statement

The study and all experiments were approved by the “Ethic committee of Auvergne (No.C2EA-02, France) for the use of Animals in Research” (Comité d'Ethique pour l'Experimentation Animale Auvergne). The protocol has not been attributed any license number since experiments have been conducted before 2013 (Décret n°2013–118 du 1^er^ février 2013 relatif à la protection des animaux utilisés à des fins scientifiques).

### Animals

Seven weeks old Albino Sprague-Dawley rats, equivalent numbers of male and female, were used in the present study. They were raised in dim-cyclic-light (12 hours dark/12 hours light; < 15 lux), fed *ad libitum* (A04, SAFE, Canada) and had free access to water. Daily observation of the animals has been done during the all experiments; no sign of suffering was noted.

### Treatment

Rats were orally fed daily by using a gastric canula with 0.2 ml of water or dietary supplement. Dietary supplement composition and daily dose (mg/rat/day) are given in Table 1.

**Table 1:** Dietary supplement composition.

Ingredients	mg/rat/day
*Vitamins and trace elements*	
Vitamin C	30
Vitamin E	5
Zinc (sulfate)	2.5
Copper (sulfate)	0.16
*Essentials Fatty Acids*	
Fish oil	116
With 70% omega-3	81.16
- EPA	46.33
-DHA	23.16
-DPA	5.83
*Extract of Tagetes Erecta*	
Lutein	1.66
Zeaxanthin	0.33
*Extract of Vitis Vinifera*	
Resveratrol	0.16

### Progressive Light Damage (PLD)

Rats were transferred for one week to bright cyclic light (12 hours dark/12 hours light) set at 400 lux (Photometer S350; United Detection Technologies, Hawthorne, CA). There were two rats per cages and they had free access to food and water.

### Anesthesia

Rats were anesthetized by a mixture of ketamine (Clorketam1000; Vetoquinol, France) and xylazine (Sigma Aldrich; St. Quentin Fallavier, France) at 150 mg/kg and 6 mg/kg, respectively.

### Electroretinography

Animals were dark adapted overnight. Under dim-red-light, they were anesthetized and their pupils dilated with 1 drop of Mydriaticum (Rhône-Mérieux, France). Rats were placed on a temperature-regulated heating pad throughout the recording session. A photostimulator (Type PS 33, Grass, USA) and neutral density filter to attenuate luminance were used to generate ElectroRetinoGram (ERG). Strobe flash ERGs (10 μs) were recorded using an Ag/AgCl electrode in contact with the corneal surface. An Ag//AgCl electrode was placed on the tong and a copper reference screen under the animal. Dark-adapted responses were presented within an integrating sphere (Labsphere, France) that mimics a ganzfeld and allow to illuminate uniformly the all retina. Single ERG was recorded for seventeen increasing luminance (10 μs duration) using flash intensities ranging from -3.47 to +0.46 log (cd.s.m^-2^). Conversely, the duration of the interstimulus interval is 30 s since this interval has been shown to be sufficient for a flash not to alter the next flash response. The signal was amplified (gain 1000, pass band 0.1–10000 Hz; A-M systems, Inc; Model 3000. AC/DC Differential Amplifier) then averaged and stored in a computer. Intensity—response functions were obtained in a single session.

#### ERG analysis

The leading edge of the a-waves obtained in response to high-intensity stimuli was analyzed with Eq 1, a modified form of the Lamb—Pugh model of rod phototransduction [15–17]:

$$\text{P3 }==\;\left\{1-\text{exp}\left[-\text{iS}{\left(\text{t}-\text{td}\right)}^{\text{2}}\right]\right\}{\text{A}}_{\text{max}}$$ (1)

where P3 represents the massed response of the rod photoreceptors and is analogous to the PIII component of Granit [16]. The amplitude of P3 is expressed as a function of flash energy (*i*) and time (*t*) after flash onset. *S* is the gain of phototransduction, A_max_ is the maximum response, and *t*_d_ is a brief delay.

The amplitude of the b-wave is calculated from the minimum of the a-wave to the maximum of the b-wave. Intensity—response function of the b-wave amplitude was fitted with the Naka—Rushton equation:

$$B\text{/}B_{\text{max}}{\;=\text{ I}}^{n}\text{/}\left({\text{I}}^{n}{\text{+}K}^{n}\right)$$ (2)

where *I* is the stimulus luminance of the flash (2,88 cd.s.m^-2^); *B* is the b-wave amplitude of ERG at *I* luminance; *B*_max_ is the asymptotic b-wave amplitude; *K* is the half-saturation constant, corresponding to retinal sensitivity; and *n* is a dimensionless constant controlling the slope of the function. The latency is the time interval between the stimulation and the peak of the b-wave or the a-wave.

### Rod response recovery

To evaluate the rod response recovery after bleaching, a single test flash of 2.88 (cd.s.m^-2^) was presented on dark-adapted retina, and then rats were exposed to a steady light for 2 min to bleach the rods. Immediately after bleaching and then every 10 min for 90 min, a single test flash of 2.88 (cd.s.m^-2^) was presented. The a-wave response at the indicated time after bleaching was normalized to the initial dark-adapted response for each rat.

### Histology and Apoptotic cell detection

Eyes were embedded in paraffin, as described previously [9]. Sections (5 μm) were cut along the vertical meridian through the optic nerve. Outer nuclear layer (ONL) thickness was measured every 0.36 mm from the optic nerve to the inferior and to the superior ora serrata. Area under the curve was integrated with the use of software (Microsoft Origin 6.0; Microcal Software, Northampton, MA). The apoptosis detection kit (Apoptag S7101; Qbiogen, Ilkirch, France) was used in accordance with the manufacturer’s instructions on 5 μm sections cut along the meridian through the optic nerve. Positive cells were counted under a microscope at 1.17 mm and 2.34 mm from the optic nerve in the superior and inferior part of the retina on a 360 μm section length.

### Fatty acid composition in plasma and retina

Rats were anesthetized before cervical dislocation. Blood samples were obtained by cardiac puncture, centrifuged at 3000g in EGTA-containing tubes for 10 min at 4°C. The neural retinas were dissected from the eye, as described previously [18]. Two retinas from one animal were homogenized in PBS-1X and centrifuged at 10000g for 10 min at 4°C. Samples were conserved at -80°C until fatty acid extraction.

#### Total lipids extraction

Total lipids were extracted from plasma and retina samples using chloroform/methanol (2:1, v/v) with 0.5% butylated hydroxytoluene (BHT) as an antioxidant [19]. Subsequently, total lipids were separated into non polar lipids or neutral lipids and polar lipids or phospholipids by solid-phase extraction (Sep-Pak, vac 1 cc, 100 mg; Waters, Guyancourt, France) as described by Juaneda P et al. [20]. Briefly, the solid phase extraction cartridges were washed with chloroform (4 ml) to elute neutral lipids followed by 8 ml methanol to elute polar lipids (phospholipids).

#### Total lipids analysis

Total phospholipids from neural retina and plasma were evaporated to dryness under a gentle stream of N_2_ to minimize oxidation. Then total phospholipids were dissolved in 200 μl methanol and 100 μl toluene for methylation. Phospholipids fatty acid methyl esters (FAMEs) were obtained after trans-esterification with 50 μl of sodium methoxide in methanol (Sigma-Aldrich, St Louis, MO, USA) followed by acid trans-esterification with 500μl of boron trifluoride in methanol (14%, Sigma-Aldrich). Before analysis, FAMEs and dimethylacetals (DMAs, from plasmalogen type) were subsequently extracted with hexane, evaporated, and diluted in 200 μl hexane and stored at—80°C.

#### Gas chromatogram analysis

The profile of total FAMEs was established by Gas-liquid chromatography using a gas chromatograph GC Trace (Thermo Fischer Scientific, Courtaboeuf, France), equipped with a fused silica CP-Sil 88 capillary column (100% cyanopropyl-polysiloxane, 100 m, 0.25 mm in inner diameter, 0.20 μm in film thickness; Varian S.A, Les Ulis, France), a programmed temperature vaporisation injector (250°C) and a flame-ionization detector. The sample (1μl) was injected in the splitless mode. The oven temperature programme ran between 70 and 225°C in four separate steps. He gas was used as a carrier, with a constant pressure (264 kPa). The identities of sample methyl esters were determined by comparing their relative retention times with those of external well-known FAME standards (Supelco 37 Component Fatty Acid Methyl Esters Mix and Menhaden Oil; Sigma Aldrich, St Quentin Fallavier, France). Other standard FAME mixtures were obtained from Nu-Chek-Prep (Elysian,MN, USA). The fatty acid profiles were expressed in relative amounts (% total fatty acids). The relative plasmalogen amount is calculated as a ratio of dimethylacetals (DMAs) to methyl esters.

### Rhodopsin Measurement

Under dim red light, each retina was homogenized in 450 μl of buffer containing 10 mM Tris-Hcl (pH 7.4), 150 mM NaCl, 1 mM EDTA, 2% (w/v) octylglucoside, and 50 mM hydroxylamine. Homogenates were centrifuged at 16,000 x *g*, and soluble lysates were scanned from 300 to 620 nm in a spectrophotometer (Thermo spectronic Rochester NY USA). Samples were then bleached for 15 minutes and scanned again. The difference spectra at 500 nm between pre- and post-bleached samples were used to determine rhodopsin content using a molar extinction coefficient of 42,000 M^-1^ [21]. Data are presented as rhodopsin content per retina.

### Experimental Design

**Fig 1:**
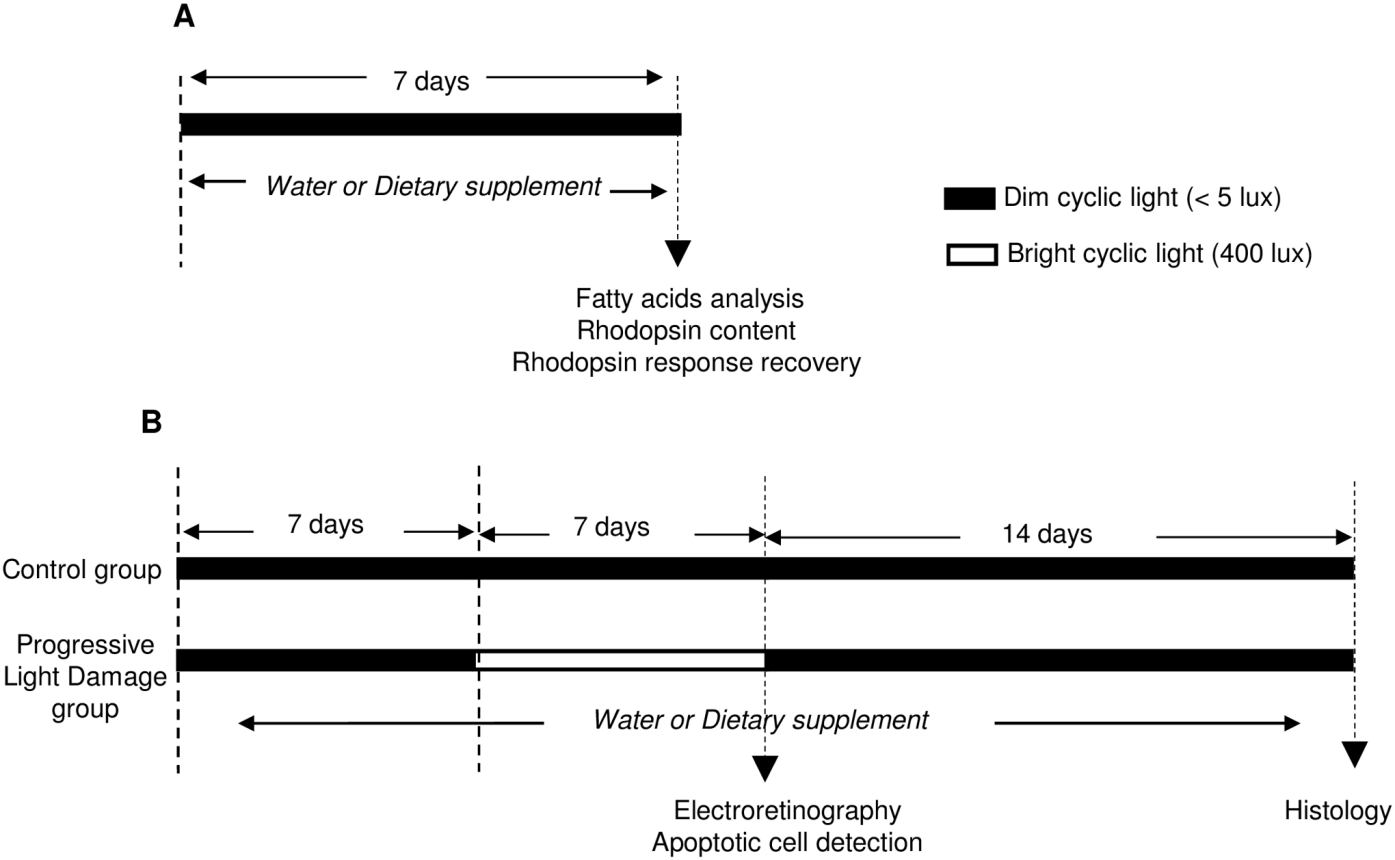
Experimental design. Rats were daily fed using a gastric canula with 0.2 ml of water or dietary supplement until they were sacrified. **(A)** In the first set of experiments (Fig 1A), after one week of treatment they were scarified for fatty acid analysis (n = 15 for water; n = 10 for dietary supplement) or rod-response recovery after bleaching (n = 10 for water; n = 11 for dietary supplement) and rhodopsin content measurement (n = 6 for each group). **(B)** In the second set of experiments (Fig 1B), one group of rats was kept in dim-cyclic-light (control group; n = 13 for water; n = 13 for dietary supplement) and the other one was transferred to bright cyclic light for one week (progressive light damage (PLD) group; 12h light at 400 lux; 12 hours dark; n = 14 for water; n = 15 for dietary supplement). At one day after progressive light damage, 8 animals from each group was used to evaluate retinal function before being sacrified to quantify apoptotic cells (n = 8 for each of group). The rest of animals (n = 6 water group; n = 7 supplemented group) were returned to dim-cyclic-light for two weeks before being sacrificed for histological analysis.

Fig 1 displays the experimental design. In the first set of experiments (Fig 1A), rats were orally fed daily with 0.2 ml of water or dietary supplement for one week in dim-cyclic-light (12 hours light < 15 lux; 12 hours dark). Then, 1/ retina and plasma were collected for fatty acids and plasmalogen analysis or 2/ rod-response recovery after bleaching was evaluated by electroretinography before collecting the retina for rhodopsin content measurement.

In the second set of experiments(Fig 1B), rats were treated with 0.2 ml of water or dietary supplement for 4 weeks: daily treatments were started one week before transferring the animals to bright cyclic light (Progressive Light Damage, PLD; 12 hours light at 400 lux; 12 hours dark) and were lasted until animals were sacrificed. At the end of the transfer, ERG was recorded (after one night of dark adaptation). Then, 8 animals per group (water and dietary supplement) were scarified for apoptotic nuclei detection and the rest (NLD: n = 5 for water and for dietary supplement; PLD: n = 6 for water and n = 7 for dietary supplement) was returned to dim-cyclic-light for two weeks before being sacrificed for histological analysis. Animals kept in dim-cyclic-light were processed in parallel.

### Statistical Analysis

Analysis of variance (ANOVA) was performed on the electroretinographic and morphometric parameters, apoptotic cell number, fatty acids and plasmalogens composition. If ANOVA was significant, multiple comparisons were made to determine which pairs of mean values were different. Significant differences between groups were assessed with the post hoc Newman-Keuls test; the significance level was set at *p* = 0.05. Significant differences between groups are noted by (*) or (†); One symbol for *p* < 0.05, two symbols for *p* < 0.01, three symbols for *p* < 0.001, and four symbols for *p* < 0.0001.

## Results

### Neuroprotective Effect

**Fig 2:**
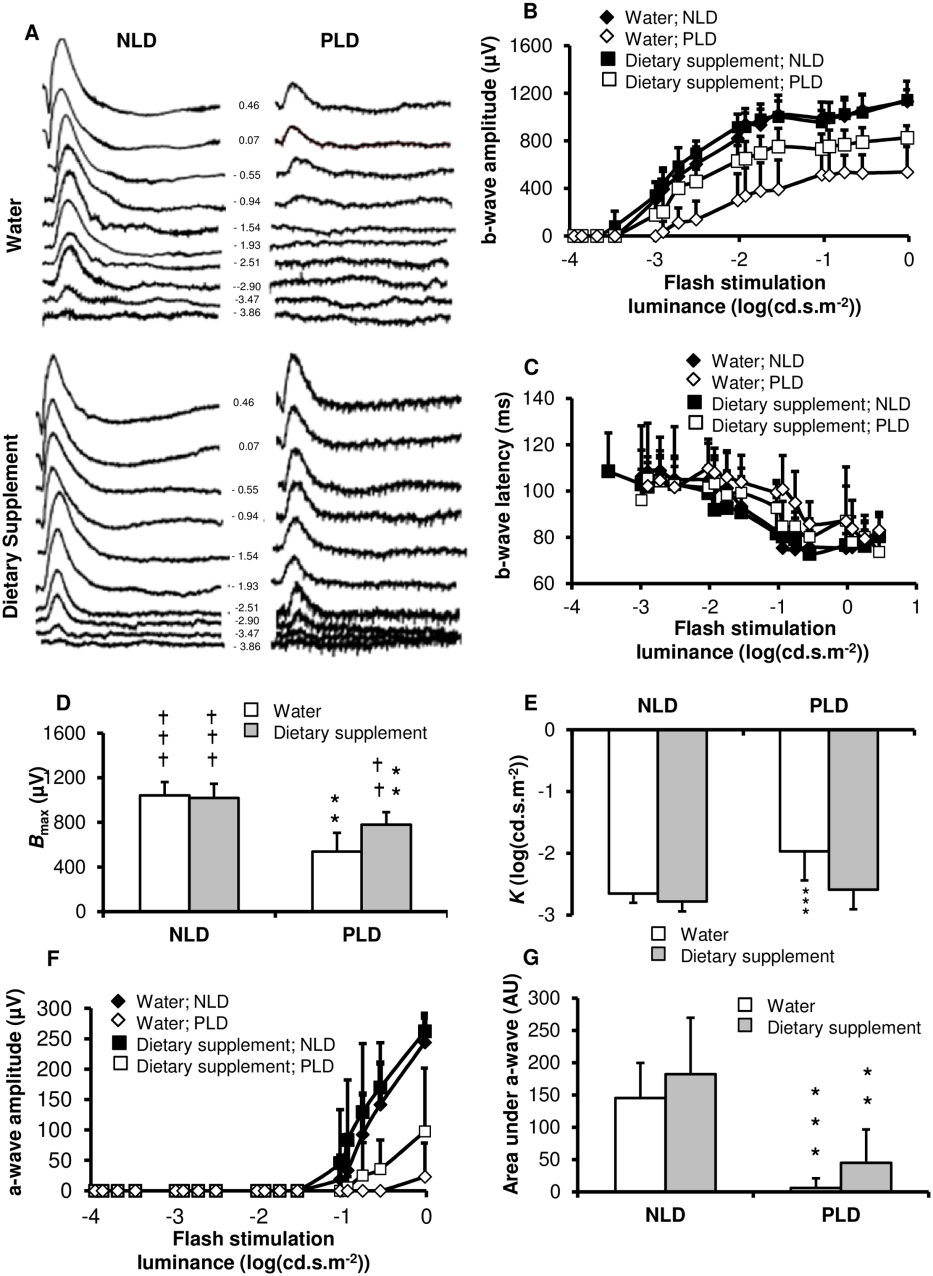
Electroretinography. Rats were daily fed using a gastric canula with 0.2 ml of water or dietary supplement for 2 weeks. After one week of treatment, rats were kept in dim-cyclic-light (No Light Damage, NLD; n = 13 for water and n = 13 for dietary supplement) or transferred for one week to bright cyclic light (Progressive Light Damage, PLD; n = 14 for water and n = 15 dietary supplement). Electroretinograms were recorded at the ends of the two weeks (**A**) **Representative dark-adapted Electroretinograms**: the numbers in the central column indicates the Flash stimulation luminance in log (cd.s.m^-2^) used to generate the Electroretinograms (ERG). (**B**) **b-wave sensitivity curve**: b-wave amplitude is plotted as a function of flash stimulation luminance. (**C**) **b-wave latency** is plotted as a function of flash stimulation luminance. The b-wave sensitivity curves are fitted to calculate the derived parameters: (**D**) ***B***_**max**_: the maximal b-wave amplitude, (**E**) ***K***: the luminance eliciting *B*_max_/2. (**F**) **a-wave sensitivity curve**: a-wave amplitude is plotted as a function of flash stimulation luminance. (**G**) **Area under the a-wave** sensitivity curve. Results are presented as Mean ± SD. (*) compared to No Light Damage and (†) compared to water-Light Damage. One symbol *p* < 0.05; Two symbols *p* < 0.01; three symbols *p* < 0.001 compared to water group.

Electroretinographic data are presented in Fig 2. Representative ERG obtained in No Light Damage (NLD) or Progressive Light Damage (PLD) animals fed with water or supplement are presented in Fig 2A. From each ERG, the b wave, a-wave amplitude as well as the wave latency was calculated. The b-wave sensitivity curves from animals kept in dim-cyclic-light (NLD; dark symbols; 12 hours light < 15 lux; 12 hours dark) were similar in supplemented and water groups (Fig 2B). There was no significant difference in the maximal b-wave amplitude (*B*_max_; 1018 ± 128 μV and 1042 ± 119 μV, respectively; Fig 2D) or half-saturation luminance (*K*; -2.8 ± 0.2 log(cd.s.m^-2^) and -2.7 ± 0.2 log(cd.s.m^-2^), respectively; Fig 2E). In addition there was no significant difference in the b-wave latency (Fig 2C). These results show that the daily supplementation enriched with antioxidant and fish oil did not affect retinal function.

Animals that had been transferred for one week to bright cyclic light (Progressive Light Damage, PLD; 12 hours light at 400 lux; 12 hours dark; open symbols) had a collapse of the b-wave sensitivity curve. This collapse was more important in the water than in the supplemented animals. *B*_max_ was reduced (*p* = 0.0002) to 538 ± 169 μV with an increase (*p* = 0.002) in *K* to -2.0 ± 0.5 log (cd.s.m^-2^) in the PLD-water group compared to the NLD-water group. In PLD-supplemented group, *B*_max_ (780 ± 112 μV) was lower (*p* = 0.002) than in NLD one but higher (*p* = 0.002) than in the PLD-water group and there was no significant variation in *K*.

Light damage induced a significant collapse of the a-wave sensitivity curve with a reduction of the area under the curve to 6 ± 14 arbitrary units in the water group (Fig 2G). Although, the a-wave sensitivity curve of the supplemented group (area = 45 ± 52 arbitrary units) was above the one from the water group, there was no significant difference in the area under the curves.

**Fig 3:**
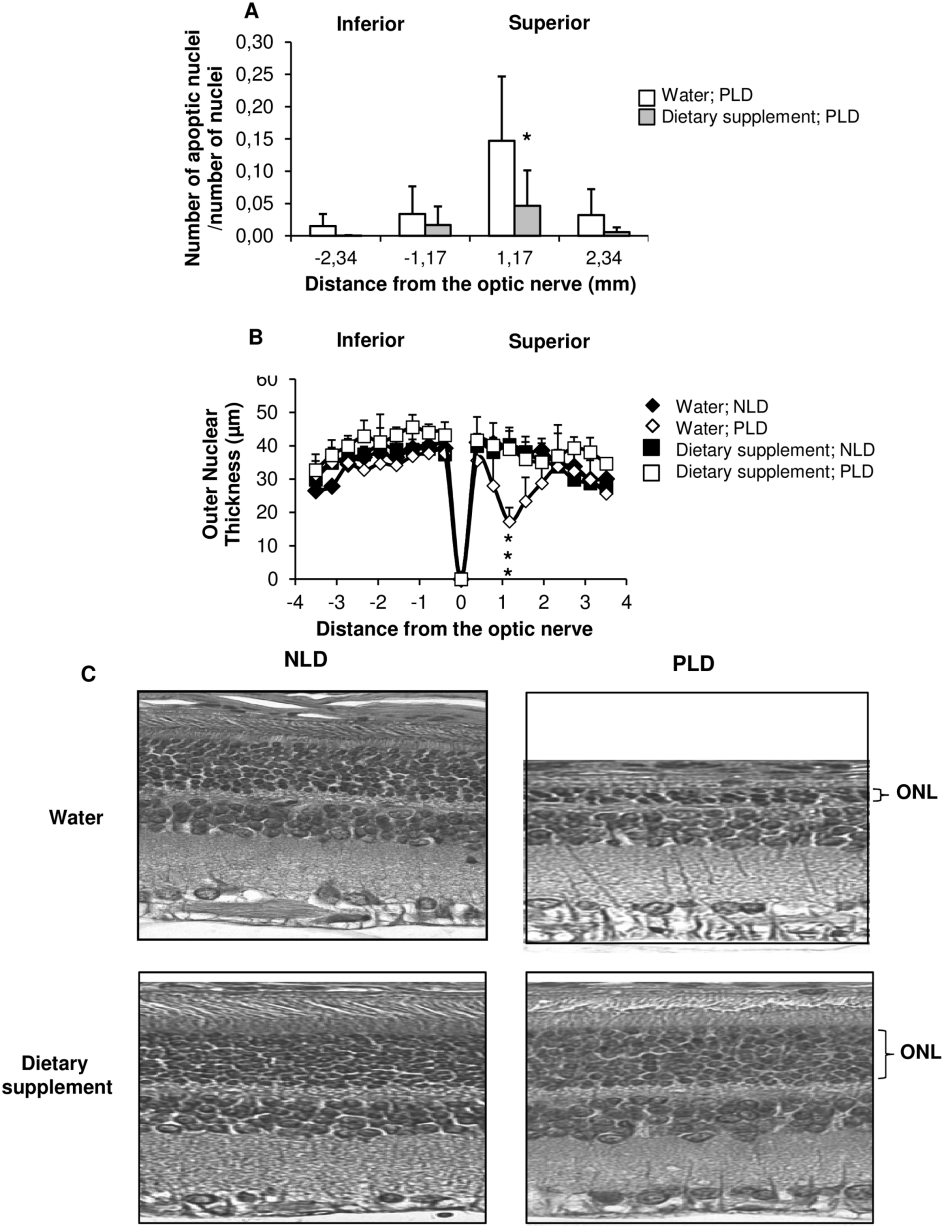
Histology and apoptotic cell detection. Rats were fed by using a gastric canula with 0.2 ml of water or dietary supplement for 4 weeks. After one week of treatment, they were kept in dim cyclic light (< 5 lux; No Light Damage, NLD) or transferred for one week to bright cyclic light (Progressive Light Damage, PLD; 12 hours light at 400 lux; 12 hours dark). 8 animals per group (water and dietary supplement) were sacrified for apoptotic cell detection and the rest (PLD: n = 6 for water and n = 7 for dietary supplement) was returned to dim cyclic light for two weeks before being sacrified for outer nuclear layer (ONL) thickness measurement. A) Number of apoptotic nuclei detection: The ratio of the number of apoptotic nuclei over the number of nuclei was calculated at 1.17 and 2.34 mm from the optic nerve in the superior and inferior side of the retina (n = 8 per group). (*) significance compared to water group. Results are presented as mean ± SD. * *p* < 0.05; ** *p* < 0.01; *** *p* < 0.001. (B) Outer Nuclear Layer thickness: The ONL thicknesses were measured from the optic nerve to the superior and inferior side of the retina (NLD: n = 5 for water and for dietary supplement; PLD: n = 6 for water and n = 7 for dietary supplement). (*) significance compared to No Light Damage group. (C) Representative micrographs of the most damaged area in the superior retina.

In order to evaluate the number of photoreceptors in the retina, we have measured the outer nuclear layer (ONL) thickness along the retina in water or supplemented animals that have been transferred or not to the PLD for one week. The ONL thicknesses were plotted as a function of the distance from the optic nerve (Fig 3B). In the NLD animals, the ONL thicknesses were similar between water and supplemented animals. The ONL thickness was 40 ± 6 μm in the water-group and 40 ± 5 μm in the supplemented group at 1.17 mm from the optic nerve. Transferring the animals to PLD has induced thinning of the superior part of the retina. The major damage area at 1.17 mm from the optic nerve was reduced (*p* = 0.002) to 17 ± 4 μm in the PLD-water group (Fig 3B and 3C). PLD-supplemented animals had an ONL thickness preserved. At 1.17 mm from the optic nerve, the ONL was thicker (*p* = 0.0002) in PLD-supplemented animals (39 ± 5 μm) than PLD-water one and was not significantly different from the two NLD group (water and dietary supplement). Therefore, the daily supplementation enriched with antioxidant and fish oil protected retinal structure from progressive light damage.

The ratio of the number of apoptotic nuclei over the number of nuclei in the ONL is presented in Fig 3A. This ratio was lower in PLD-supplemented than in PLD-water retinas whatever the considered area was. At 1.17 mm from the optic nerve in the superior part of the retina, the ratio was reduced (*p* = 0.02) from 0.14 ± 0.09 apoptotic nuclei/nuclei in water group to 0.04 ± 0.05 in the supplemented group. Therefore, the daily supplementation enriched with antioxidant and fish oil reduced photoreceptor cells apoptosis.

### Fatty acids and plasmalogens

**Fig 4:**
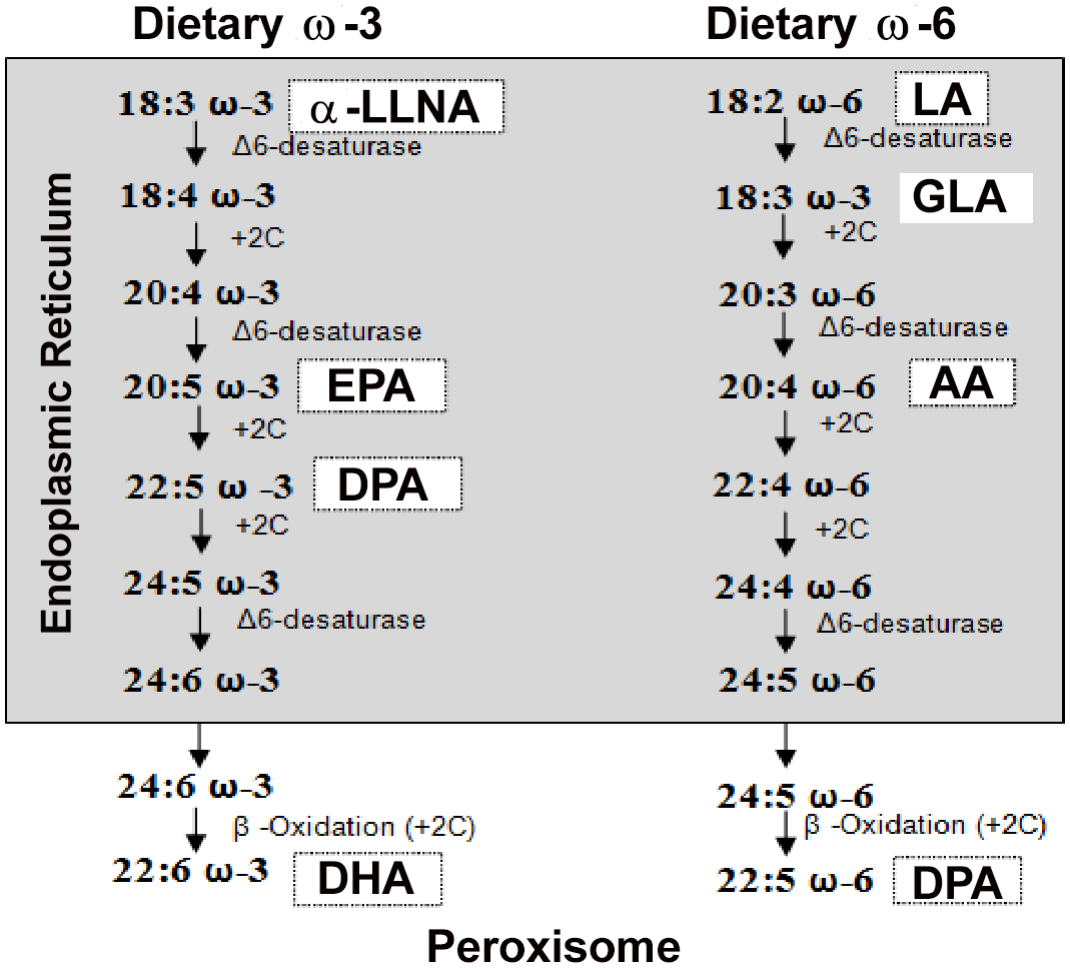
Biosynthetic pathways of omega-3 and 6 fatty acids. Fatty acid notation represents total number of carbons, number of double bonds, and position of the first double bond relative to the methyl terminal of the hydrocarbon chain. For example, 22:6 ω*-3* indicates that the fatty acid chain is 22 carbons long with the first of 6 double bonds inserted between the third and fourth carbons from the methyl terminal. α- LLNA = α-linolenic acid, EPA = eicosapentaenoic, DHA = docosahexaenoic acid, LA = linoleic acid, AA = arachidonic acid, DPA = docosapentaenoic acid, GLA = gamma-linolenic acid.

In the retina, phospholipids represent about two-thirds of total lipids in the structure and are characterized by species rich in Polyunsaturated Fatty Acids (PUFAs). The most abundant PUFA is DHA which belongs to the omega-3 family. The reduced level of DHA in plasma and in photoreceptor cells in the retina is a characteristic feature of retinal degenerations such as AMD [22] or Retinis Pigmentosa (RP) [23]. The synthesis of omega-3 fatty acids is in competition with those of omega-6 family. Fig 4 displays biosynthetic pathway of the omega-3 and omega-6 families. Plasmalogens constitute specific phospholipids and are expressed in the retina (in the inner segment of photoreceptors) [24]. Studies have suggested that one of the biological functions of plasmalogens (Pls) is to protect animal cell membranes against oxidative stress [25–27].

In order to determine the effect of daily supplementation (enriched with antioxidant and fish oil) on fatty acids and plasmalogens, we have analyzed plasma and retina by gas chromatography the first week of treatment.

**Fig 5:**
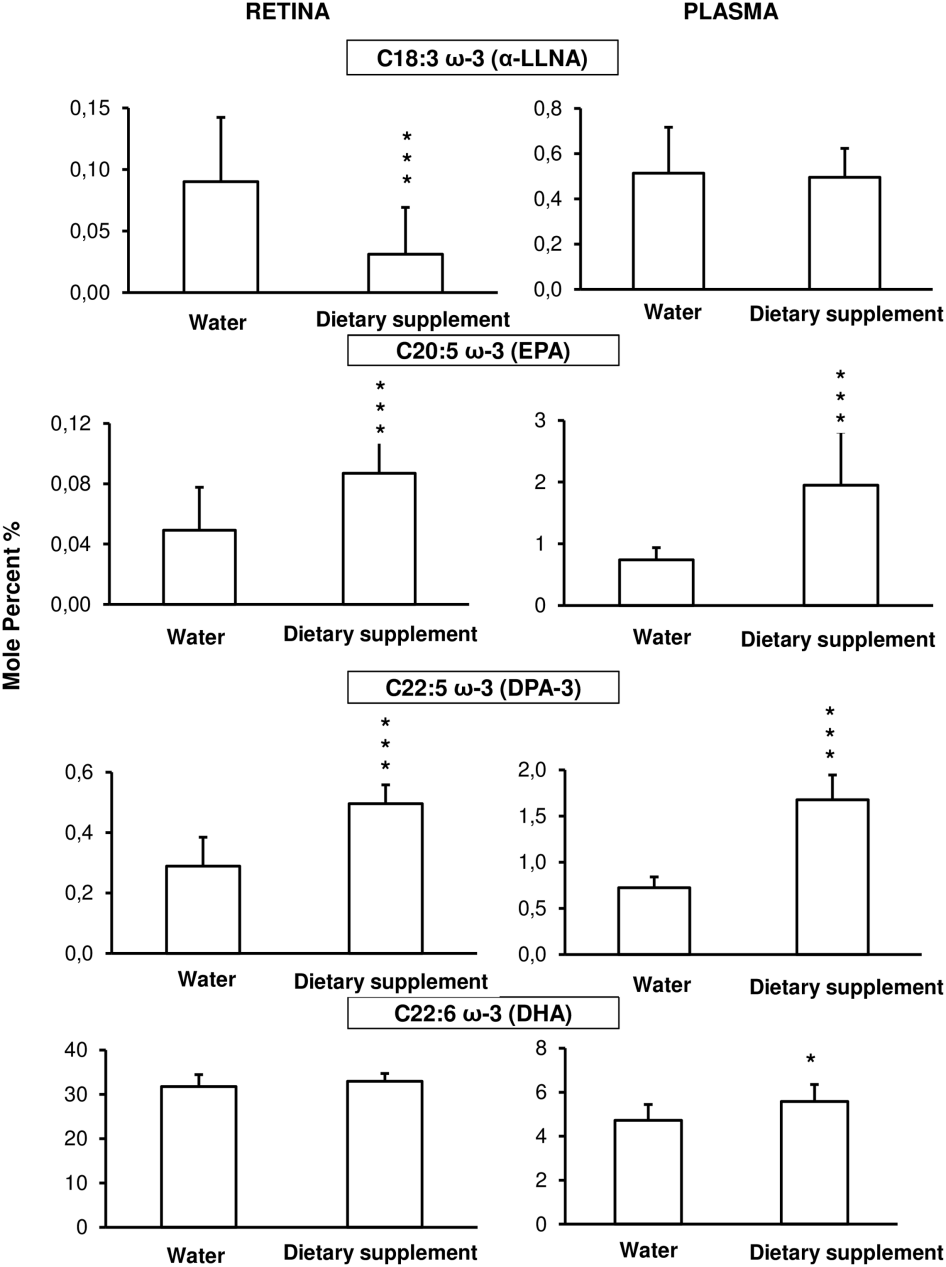
Omega-3 fatty acid composition in plasma (right column) and retina (left column). Animals were daily fed by using a gastric canula with 0.2 ml of water (n = 15) or dietary supplement (n = 10) for one week before the fatty acid analysis. Results are presented as percentage ± SD of the total fatty acid. * *p* < 0.05; ** *p* < 0.01; *** *p* < 0.001 compared to water fed by gavage group.

**Fig 6:**
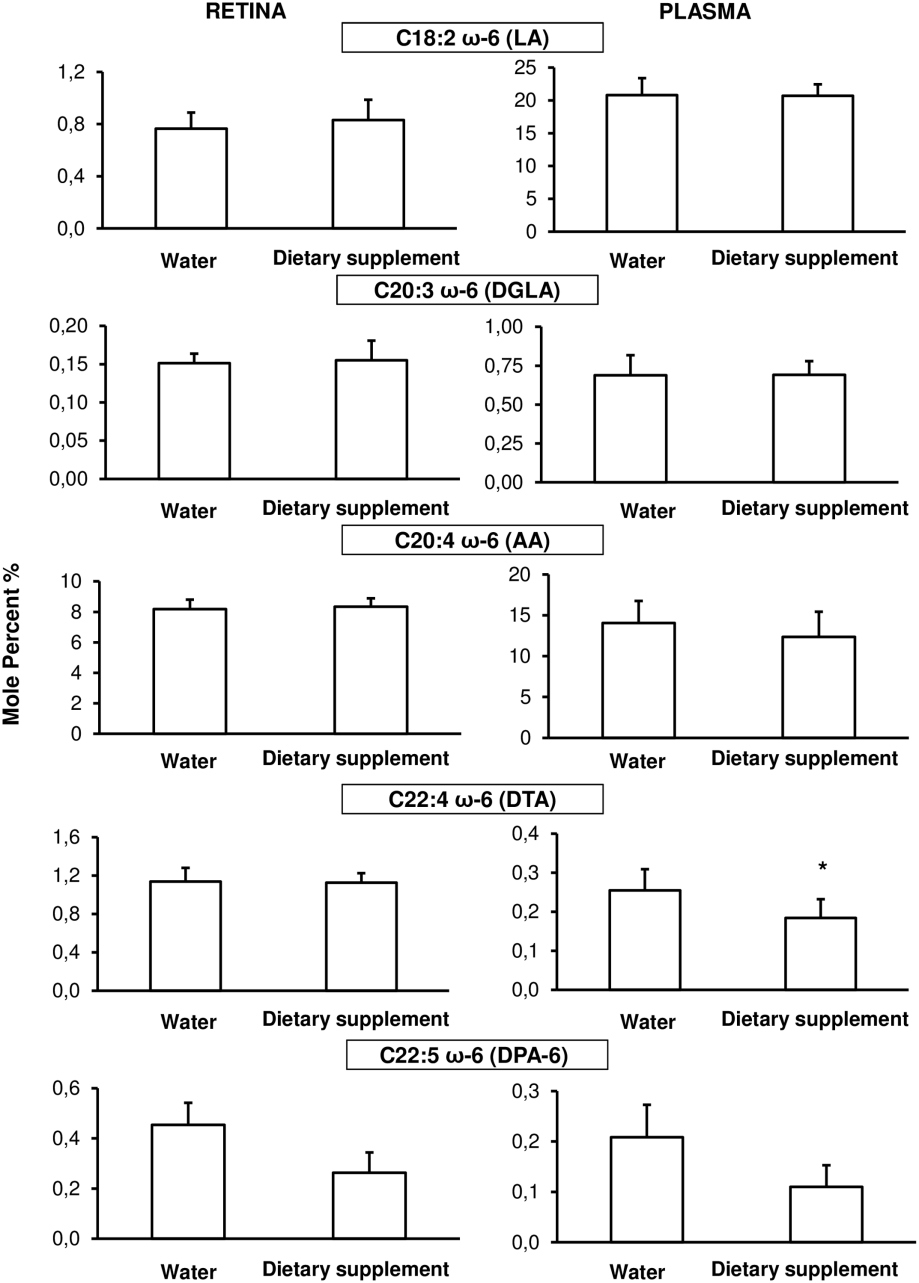
Omega-6 fatty acid composition in plasma (right column) and retina (left column). Animals were daily fed by using a gastric canula with 0.2 ml of water (n = 15) or dietary supplement (n = 10) for one week before the fatty acid analysis. Results are presented as percentage ± SD of the total fatty acid. * *p* < 0.05; ** *p* < 0.01; *** *p* < 0.001 compared to water fed by gavage group.

In plasma, the level of omega-3 precursor (α-linolenic acid, α-LLNA, C18:3ω-3, Fig 5) and the omega-6 precursor (linoleic acid, LA, C18:2ω-6, Fig 6) was not significantly different between water and supplemented animals. There was an increase in omega-3 eicosapentaenoic (EPA, C20:5ω-3; *p* = 0.003); docosapentaenoic (DPA-3, C22:5n-3; *p* = 0.0002) and docosahexaenoic (DHA, C22:6ω-3, *p* = 0.035) in plasma from supplemented (1.95 ± 0.9%; 1.7 ± 0.3% and 5.6 ± 0.7%, .respectively) compared to water (0.7 ± 0.2%, 0.7 ± 0.3 and 4.7 ± 0.8%, respectively) animals (Fig 5). In the omega-6 fatty acids (Fig 6), gamma-linolenic acid (GLA, C18:3ω-6), dihomo-gamma-linolenic acid (C20:3ω-6) or arachidonic acid (AA, C20:4ω-6) did not vary significantly in plasma from supplemented compared to water animals, but there was a decrease in docosatrienoic acid (DTA, C22:4ω-6; *p* = 0.012) and in docosapentaenoic acid (DPA-6, C22:5ω-6; *p* = 0.003) by 45% and 28% respectively.

In the retina, α-LLNA was decreased (*p* = 0.004) in supplemented (0.03 ± 0.01%) compared to water (0.09 ± 0.05%) animals (Fig 5). The omega-3 EPA and DPA were higher (*p* < 0.005) in supplemented (0.09 ± 0.03% and 0.50 ± 0.06%, respectively) than in water (0.05 ± 0.03% and 0.29 ± 0.09%, respectively) retinas. There was no significant difference in DHA level. The only omega-6 fatty acid affected by the treatment was DPA which was lower (*p* = 0.012) in supplemented (0.26 ± 0.08%) than in water retinas (0.45 ± 0.09%) (Fig 6).

**Fig 7:**
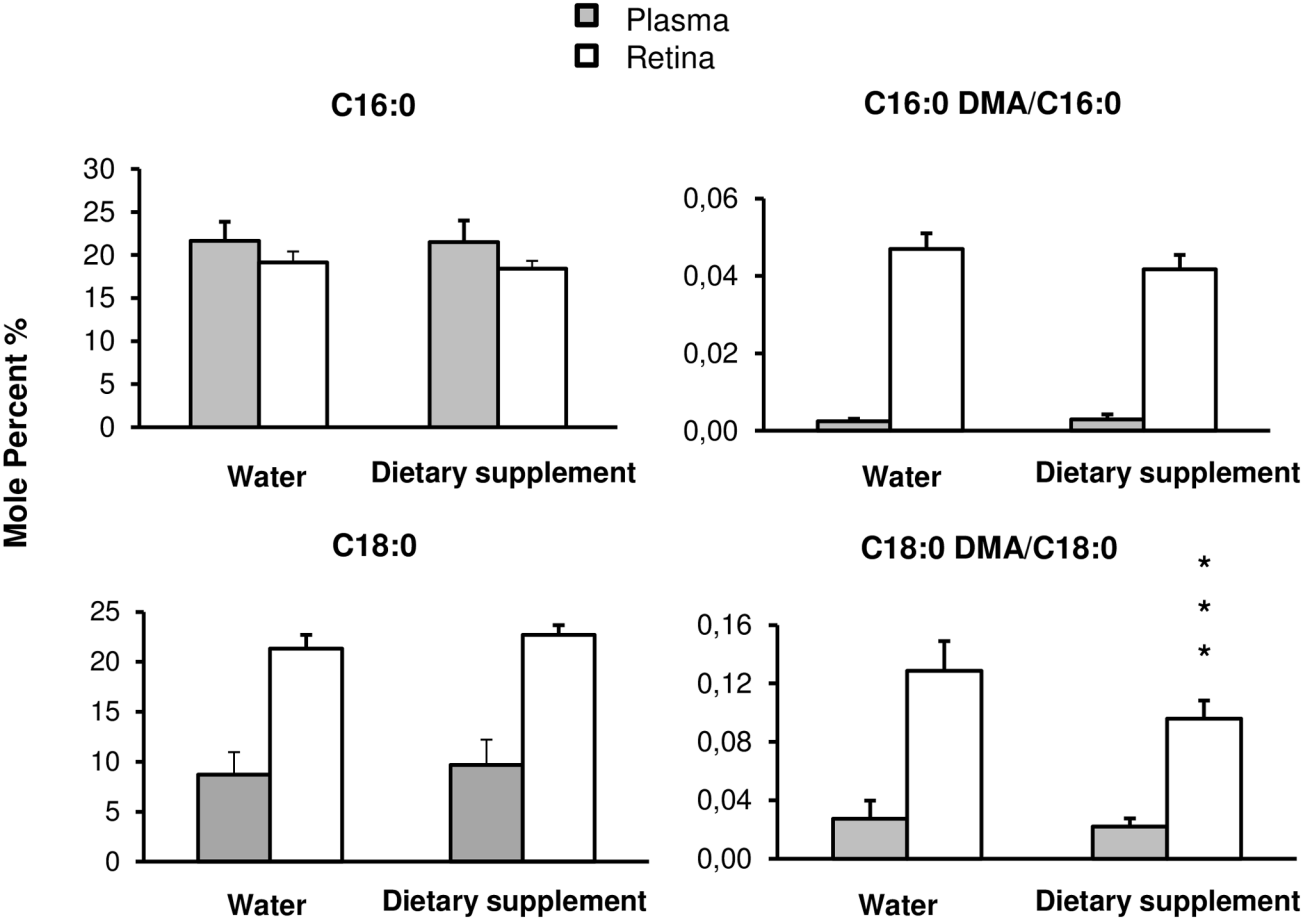
Plasmalogens. Animals were daily fed by using a gastric canula with 0.2 ml of water (n = 15) or dietary supplement (n = 10) for one week before plasmalogens analysis. In Plasmalogen, the aliphatic moieties at the *sn*-1 position consist of Palmitic Acid (C16:0) or Stearic Acid (C18:0) and Plasmalogen ratio is calculated as C18:0 DMA/C18:0 and C16:0 DMA/ C16:0 in plasma and retina. DMA: dimethyl aldehyde. Results are presented as percentage ± SD of the total fatty acid. * *p* < 0.05; ** *p* < 0.01; *** *p* < 0.001 compared to water fed by gavage group.

The relative plasmalogens amount was calculated as a ratio of dimethylacetals (DMAs, from plasmalogens type) to methyl esters. Dimethyl aldehyde stearic acid (DMA C18:0), dimethyl aldehyde palmitic acid (DMA C16:0), Stearic acid (C18:0) or palmitic acid (C16:0) was not significantly different between groups in plasma (Fig 7). In retina, there was an increase (*p* = 0.01) in C18:0 from 21 ± 1% in water to 23 ± 1% in supplemented animals and a reduction (*p* < 0.003) in C18:0 DMA and C16:0 DMA leading to a relative amount of (C18:0 DMA/C18:0) and (C16:0 DMA/C16:0) lower (*p* < 0.009) in supplemented (0.042 ± 0.004% and 0.10 ± 0.01%, respectively) than in water (0.047 ± 0.004% and 0.13 ± 0.02%, respectively) retinas.

### Rhodopsin and rod response recovery

**Fig 8:**
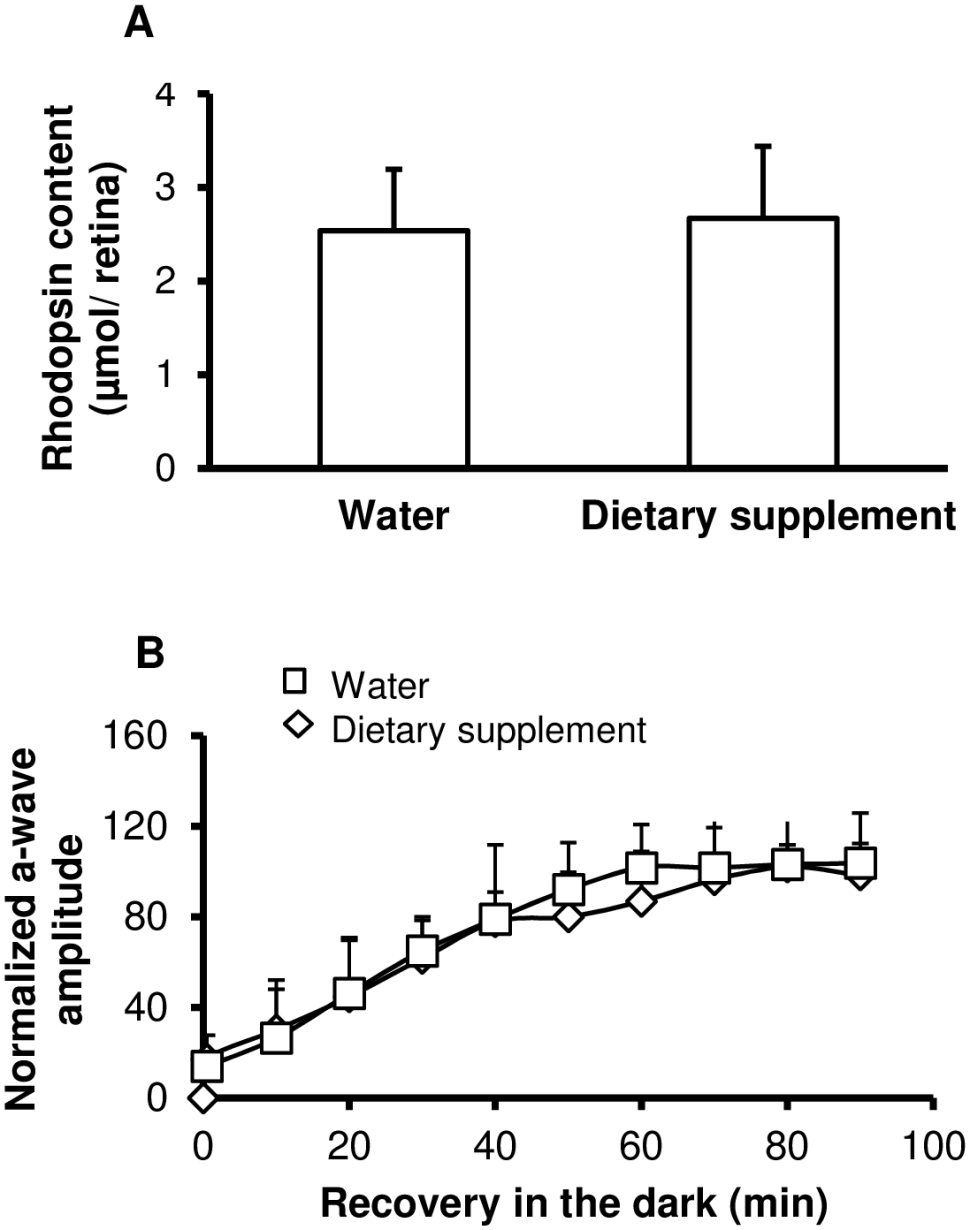
Rhodopsin content and regeneration. (A) Rhodopsin content in the retina of rats fed using a gastric canula with 0.2 ml of water (n = 6) or dietary supplement (n = 6) for one week. Results are presented as mean value ± SD μmol of rhodopsin/eye. (B) Rod-response recovery. Electroretinograms were recorded from water (diamond, n = 10) and dietary supplement (square, n = 11) rats. A single test flash of 2.88 (cd.s.m^-2^) was presented on dark-adapted rats. Rats were then exposed to a steady light for 2 min to bleach the rods. Immediately after bleaching and then every 10 min for 90 min, a single test flash of 2.88 (cd.s.m^-2^) was presented. The a-wave responses at the indicated times after bleaching were normalized to the initial dark-adapted response for each rat. The error bars represent ± SD.

Because rhodopsin content or rhodopsin regeneration through the visual cycle can affect retinal sensitivity to light [21], we have measured rhodopsin content (Fig 8A) in the retina and rod-response recovery by electroretinography (Fig 8B) after one week of water or dietary supplementation. Rhodopsin content was not significantly different between water (2.24 ± 0.33 μmoles/retina) and supplemented (2.57 ± 0.22 μmoles/retina) groups and rod-response recovery was not significantly affected by supplementation. These results indicate that the daily supplementation enriched with antioxidant and fish oil did not affect rhodopsin content or regeneration.

## Discussion

Although, light is essential for vision, it can initiate pathological processes within visual cells commonly referred as retinal light damage and it gives rise to oxidative damage, inflammation and apoptotic reactions leading to photoreceptor cell death. Oxidative stress has been implicated in retinal light damage processes and it has been postulated as a risk factor associated with the initiation and/or progression of retinal degeneration in Age-Related Macular Degeneration(AMD) [28] and some form of inherited retinal degeneration [29]. One of the therapeutic strategies to prevent or at least slow down development or progression of retinal degeneration consist of improving retinal antioxidant capacities with dietary supplement. Based on Age-Related Eye Disease study 1 and 2 (AREDS 1 and AREDS 2), several different ocular dietary supplements have been developed and are already on the market without safety and efficacy approval. In the present study, we have investigated one of these dietary supplements, for the first time, in an in-vivo model of light-induced retinal degeneration.

The dietary supplement used in the present work differs from the formulations used in AREDS 1 and AREDS 2. It contains the macular carotenoids lutein and zeaxanthin as used in AREDS 2 but not β-carotene. Evidence has accumulated that lutein/zeaxanthin have potent radical scavenger as well as antioxidative properties and, indeed, secondary analysis of AREDS 2 indicates that supplementation is beneficial in AMD [5,30]. In addition, omega-3 free fatty acids are part of the dietary supplement used in our study, with more eicosapentaenoic acid (EPA) than docosahexaenoic acid (DHA) in contrary to AREDS 2. Omega-3 free fatty acids have a key role in determining the permeability, fluidity, thickness, and lipid phase of photoreceptor membranes. DHA content has been shown to be reduced in outer segment of photoreceptors that are fated to degenerate [31,32] and in blood during retinal degeneration [33–35] leading to the hypothesis that photoreceptor fatty acid content and mainly omega-3 content might be an important factor involved in retinal degeneration. In addition, DHA is the precursor for neuroprotectin D1 which displays a potent neuroprotective bioactivity [36]. For its part, EPA can compete with the production of pro-inflammatory eicosanoids from arachidonic acid (omega-6) by generating anti-inflammatory eicosanoids [37]. The relative dose of zinc in the present supplement was reduced compared to AREDS 1 and even compared to the lower dose tested in AREDS 2. Moreover, the dietary supplement tested, also contained an antioxidant that was not part of the spectrum tested in AREDS 1 and 2, resveratrol. Resveratrol, one of dietary polyphenol found in grapes, nuts and other plants, is known to prevent retinal degeneration related to light damage [38,39]. The first important result obtained in the present study was that the dietary supplement was not toxic over a period of daily gavage of 4 weeks.

Whereas most of studies using light damage were conducted by exposing the animals to short-term illumination (2 hours to 24 hours) with high light intensity (1000–8000 lux) [40–42], in the present study we have used a model consisting in exposing the animals for one week to 400 lux of cyclic light (12 hours light/12 hours dark) leading to progressive light-induced retinal degeneration (PLIRD). Retinal degeneration is characterized by a loss of photoreceptors cells by apoptosis, a transitory alteration of the surviving photoreceptors as shown by the transient increase in the half-saturation luminance (*K*) that finally results in a 48% loss of function. These results were in agreement with others who showed that light damage induced photoreceptor apoptosis [43,44] or that an increase in cyclic light intensity from 15 to 750 lux resulted in substantial decreases in photoreceptor cell densities with alteration of the surviving photoreceptors [45].

In the present study, the dietary supplement tested has prevented retinal structure and function loss from the cumulative damaging effect of light. It is important to mention that the slight reduction in retinal function still observed in the supplemented group after PLIRD was not due to photoreceptor loss since the thickness of the outer nuclear layer was not affected by PLIRD confirming that the photoreceptors are preserved. This reduction is likely due to an adaptive mechanism to the bright environmental light condition compared to dim-cycling rearing condition. Indeed, it is well known that in bright cyclic light rearing condition the outer segments are shorter and rhodopsin packing is reduced [46]. In order to confirm this hypothesis experiments are on course to evaluate rhodopsin content and rod ultra-structure in supplemented animals exposed to PLIRD compared to the one in dim-cyclic-light.

Thereafter, to better understand the protective mechanism, we have evaluated for the first time, retinal and plasmatic fatty acids and plasmalogens changes induced in-vivo by a complex supplement. Whereas most studies have evaluated fatty acid level after a long period of dietary manipulation (1–3 months) [47,48], we have looked at only after one week. DHA was increased significantly in plasma but in the retina its level was not changed. These findings are in agreement with the fact that DHA handling and trafficking by the retina is specifically orchestrated around a conservation mechanism that ensures adequate levels of DHA for photoreceptors at all times [49]. EPA and docosapentaenoic acid (DPA), the two previous steps in the biosynthetic pathway to DHA, were increased in plasma and retina of supplemented animals. As expected, the increases in omega-3 fatty acids were associated with a lower level of omega-6 fatty acids, omega–6 polyunsaturated fatty acids (PUFAs) biochemically competing with omega–3 PUFAs [37,50,51]. Dietary supplement had no effect on the level of α-linolenic acid (α-LLNA), the omega-3 precursor, in plasma. But α-LLNA was largely reduced in the retina. These data suggest a decrease in α-LLNA incorporation from the circulation since the blood supplies directly EPA and DHA. This hypothesis is supported by the fact that the tissue accretion is highest when the Long Chain PUFAs are ingested in the preformed state [52]. Therefore, our hypothesis is that the beneficial effect of the dietary supplement used in the present study is in part due to this accumulation of EPA. EPA storage can (1) facilitate DHA supply from neo-synthesis in the retina or (2) compete with the production of pro-inflammatory eicosanoids from arachidonic acid (omega-6) by producing less inflammatory or even anti-inflammatory eicosanoids [37]. However, it is important to remember that the other molecules supplied by the dietary supplement used can interact within the retina and/or retinal pigment epithelium in a way to optimize neuroprotection [53,54].

As for other tissues or cell types, retinal phospholipids also consist of particular phospholipids called plasmalogens (Pls), which are likely to be synthetized in the inner segment of photoreceptor cells, and in the retinal pigmented epithelium (RPE) cells [55]. Pls are of particular interest since they have been described as physiological antioxidants. The vinyl ether functionality serves as a sacrificial trap for free radicals and singlet oxygen [56,57]. In addition, Pls containing at their sn-2 position arachidonic or docosahexaenoic acids, are suggested to be reservoirs of PUFAs, which are released upon proper stimulation [58]. Moreover, an increase of Pls has been reported in dominant, recessive, and isolates forms of Retinis pigmentosa patients [59]. To our knowledge, no studies have established the influence of dietary supplement on retinal and palsma Pls. In the present study, Pls level did not vary in plasma, but it decreased in the retina of supplemented animals. These results suggest that a lower level of plasmalogens is beneficial for the retina. This hypothesis is supported by the work of Stadelmann-Ingrand who has demonstrated that fatty aldehydes released from plasmalogens after oxidative stress in cerebral cortex homogenates can generate covalent modifications of endogenous macromolecules such as phosphatidylethanolamine (PE), the very reactive and toxic malondialdehyde (MDA) or 4-hydroxynonenal (4-HNE) and prevent their deleterious effects[60].

Since photobleaching and regeneration of rhodopsin have been identified as essential steps for retinal sensitivity to light damage [61,62] and rhodopsin function might be modulated by membrane composition [63], we have evaluated for the first time the effect of a dietary supplement on rhodopsin content and function. Fast regeneration of rhodopsin after bleaching increases retinal sensitivity, whereas slowing regeneration promotes photoreceptor’s resistance [21]. In the present study, we have shown that although the dietary supplement tested induced changes in retinal fatty acids composition, it did not affect rhodopsin content or rhodopsin regeneration.

## Conclusions

The present study reveals, for the first time, that dietary supplement containing lutein, zeaxanthin, vitamins C, E, Zinc, omega-3 with mainly EPA and resveratrol protects the retina from light-induced retinal degeneration without affecting rhodopsin. This indicates that the present formulation is capable of exerting anti-oxidative properties in the retina in-vivo. Therefore, this dietary supplement presents an excellent potential to be used as a preventive supplement for the progression of certain retinal disease. Experiments are on course to further investigate this neuroprotective mechanism.
